# Low HDL-cholesterol among HIV-1 infected and HIV-1 uninfected individuals in Nairobi, Kenya

**DOI:** 10.1186/s12944-017-0503-9

**Published:** 2017-06-09

**Authors:** Anne Njoroge, B. L. Guthrie, Rose Bosire, Mark Wener, James Kiarie, Carey Farquhar

**Affiliations:** 1 Department of Research and Programs, Kenyatta National Hospital, Nairobi, Kenya; 2 University of Washington, Seattle, WA USA; 3 Department of Epidemiology, University of Washington, Seattle, WA USA; 4 Department of Medicine, University of Washington, Seattle, WA USA; 5 Centre for Public Health Research, Kenya Medical Research Institute, Nairobi, Kenya; 6 Department of Medical Epidemiology and Biostatistics, Karolinska Institutet, Stockholm, Sweden; 7 Department of Obstetrics and Gynaecology, University of Nairobi, Nairobi, Kenya

**Keywords:** Dyslipidemia, Cholesterol, HIV, Kenya, HDL

## Abstract

**Background:**

Antiretroviral treatment (ART) is associated with dyslipidemia yet little is known about the burden of dyslipidemia in the absence of ART in sub-Saharan Africa. We compared the prevalence and risk factors for dyslipidemia among HIV-infected ART-naïve adults and their uninfected partners in Nairobi, Kenya.

**Methods:**

Non-fasting total cholesterol (TC) and high density lipoprotein cholesterol (HDL) levels were measured by standard lipid spectrophotometry on thawed plasma samples obtained from HIV-infected participants and their uninfected partners. Dyslipidemia, defined by high TC (>200 mg/dl) or low HDL (<40 mg/dl) was compared between HIV-infected and uninfected men and women.

**Results:**

Among 196 participants, median age was 32 years [IQR: 23–41]. Median CD4 count among the HIV-infected was 393 cells/ μl (IQR: 57–729) and 90% had a viral load >1000 copies/ml. Mean TC and HDL were comparable for HIV-infected and uninfected participants. Prevalence of dyslipidemia was 83.8% vs 78.4% (*p* = 0.27). Among the HIV-infected, those with a viral load >1000 copies/ml were 1.5-fold more likely to have dyslipidemia compared to those with ≤1000 copies/ml (adjusted prevalence ratio [aPR] 1.5, 95% CI: 1.22–30.99, *p* = 0.02). BMI, age, gender, blood pressure and smoking were not significantly associated with dyslipidemia.

**Conclusions:**

Among ART-naïve HIV-infected adults, high viral load and low CD4 cell count were independent predictors of dyslipidemia, underscoring the importance of early initiation of ART for viral suppression.

## Background

With improved access to antiretroviral therapy (ART) in sub-Saharan Africa, the life expectancy among HIV-infected individuals has increased. It is estimated that 2.2 million adults living with HIV in this region are now 50 years or older [1]. Age is traditionally associated with higher morbidity and mortality due to cardiovascular diseases (CVD) such as myocardial infarction (MI) and stroke. This risk is heightened among older HIV-infected adults [2, 3] due to HIV-specific factors, including metabolic complications associated with chronic inflammation resulting from the HIV virus itself (e.g. insulin resistance, lipodystrophy, abnormal lipid levels) and dyslipidemia resulting from ART toxicity [2].

Studies in high-income countries have demonstrated higher rates of dyslipidemia in HIV-infected individuals, both on and off ART, when compared to HIV uninfected persons, as well as higher rates of adverse cardiovascular outcomes such as myocardial infarction and stroke [4–6]. However, data on dyslipidemia and subsequent cardiac risk among HIV-infected individuals in low-income settings are limited. While studies conducted in developed countries have demonstrated high prevalence of the traditional risk factors for cardiovascular disease among HIV-infected individuals, including smoking, obesity and hypertension [7, 8], the prevalence of these factors may be different in Sub-Saharan Africa.

Several studies in SSA have described a high prevalence of dyslipidemia among HIV-infected individuals (62–87%) [9, 10] but little is known about how this compares to the prevalence of dyslipidemia among HIV-uninfected adults. While low CD4 count has been associated with increased risk of dyslipidemia in SSA, to date, the association of viral load with dyslipidemia has not been assessed as viral load testing is not routinely conducted in these settings due to limited resources [11]. It is important to assess dyslipidemia and its correlates in ART-naïve persons to prevent development of cardiovascular disease and inform the choice of subsequent ART. We therefore sought to estimate the prevalence of dyslipidemia and associated risk factors comparing ART naïve HIV-infected and uninfected individuals in a cohort of HIV-discordant couples in Nairobi, Kenya.

## Methods

### Study design and setting

We conducted a nested cross-sectional study within a parent prospective cohort study (R01 AI068431) in which ART-naïve HIV-1 serodiscordant couples were enrolled from voluntary counseling centers (VCT) in Nairobi, Kenya from September 2007 to December 2009 [12].HIV-1-infected participants with a history of clinical AIDS (WHO stage III or IV) were excluded.

In the current study, couples were divided into 2 groups: those in whom the male was HIV-infected and those with an HIV-infected female. We randomly selected 50 couples from each group. Of the individuals selected, 1 HIV-infected and 3 HIV-uninfected individuals were excluded due to inadequate plasma sample volume.

### Study procedures

The study procedures for the parent study have been described elsewhere in detail [12]. At enrollment, clinic staff administered questionnaires collecting socio-demographic data, a detailed medical history and performed clinical physical examination on participants.

Participants were tested for HIV-1 by two rapid tests conducted in parallel using a Determine HIV-1/2 rapid test (Abbott Laboratories, Tokyo, Japan) and Bioline HIV 1/2 rapid test (Standard Diagnostics Inc., Suwon, South Korea). Positive results were confirmed using an enzyme-linked immunosorbent assay (ELISA). Plasma from HIV-1-infected partners collected at enrollment was assayed for HIV-1 RNA load using Gen-Probe Transcription Mediated Amplification (Gen-Probe, San Diego, CA). This assay detects the prevalent HIV-1 subtypes in Kenya, subtypes A, C, and D and could detect as low as ~10–25 copies/ml. CD4 cell counts was measured using FACs Caliber system (Becton Dickinson). Whole blood samples were fractionated and plasma samples were stored frozen at −80 °C. Appropriate transport and thawing standards were maintained. Quantitative determination of total cholesterol (TC) and high density lipoprotein (HDL) was done using the UniCel DxC 800 (Beckman Coulter Inc.) spectrophotometry auto analyzer at the University of Washington. Ethical approval was sought and obtained from the KNH-UoN Ethics & Research Committee and the University of Washington’s IRB.

### Statistical methods

Dyslipidemia was defined as TC ≥200 mg/dl or HDL <40 mg/dl, according to the National Cholesterol Education Program Adult Treatment Panel (ATP) III guidelines [13]. The TC: HDL ratio was calculated and categorized as per the American Heart Association guidelines for CVD risk prediction: Low risk ≤3.5, intermediate 3.6–4.9 and high risk ≥5.0 [14].

Demographic, behavioral, clinical and laboratory characteristics of participants at enrollment were summarized. Age was considered both as a continuous and categorical variable grouped in 10 year categories starting from 25 years. Height and weight were measured using standardized procedures [15]. Standard Body Mass Index (BMI) categories were used. Blood pressure cut-offs defined by the European Society of Cardiology were used [16]. Socio-economic status (SES) was categorized as below or above the median monthly income per couple (Kenyan shillings).

CD4 cell count categories were defined using a cut-off of 350 cells/μl, as this was the WHO recommended cut-off for initiation of ART at the time of the study [17]. Viral load was categorized as above or below 1000 copies/ml, based on the WHO threshold defining virologic failure [17].

Counts and proportions for categorical variables as well as medians and interquartile ranges (IQR) for continuous variables were used. A 2-sample student’s t-test with unequal variance was used to compare the mean total cholesterol, HDL and TC: HDL ratio between HIV-infected and HIV-uninfected individuals. Logistic regression models were used to evaluate the association between dyslipidemia and potential predictors. All multivariate analyses were adjusted for age and gender, as a priori potential confounders. Mean plasma lipid levels did not vary upon adjustment for storage using a degradation rate previously reported in literature (1.2% - 2% for every year stored) [18]. The assessments of CD4 cell count and viral load were restricted to HIV-infected individuals. All statistical tests were evaluated using a 2-sided test with a *p*-value <0.05 to define statistical significance. Statistical analyses were conducted using Stata IC version 13 (StataCorp Inc., College Station, TX, USA).

## Results

### Description of study population

Of the 469 HIV-infected couples enrolled in the study for whom baseline data were available, 100 couples were randomly selected, of which 50% of the couples had an HIV-infected male and 50% had a HIV-infected female. A total of 99 HIV-infected and 97 HIV-uninfected individuals with frozen samples available were included in this analysis. Median age for HIV-infected participants was 32 years (interquartile range [IQR]: 24–40) and for HIV-uninfected participants the median age was also 32 years (IQR: 23–42) (Table 1). Among HIV-infected participants, 44 (44%) had normal BMI, 33 (33%) were overweight and 19 (19%) were obese while in the HIV-uninfected, 44(45%) had normal BMI, 28 (29%) were overweight and 21 (22%) were obese (*p* > 0.05 for all comparisons). For the HIV-infected group, median CD4 count was 393 cells/μl (IQR: 57–729), median plasma viral load 4.5 log_10_ copies/ml (IQR: 3.2–5.8) and 90% had a viral load greater than 1000 copies/ml. All HIV-infected individuals were ART-naïve and 92 (92%) were on Cotrimoxazole prophylaxis.

**Table 1:** Baseline characteristics of HIV-infected and uninfected individuals

Characteristic	HIV infected	HIV uninfected
	Median(IQR) or N(%)	Median(IQR) or N(%)
	*n* = 99	*n* = 97
Age(years)		
< 25	10 (10)	11 (11)
25–34	58 (58)	47 (48)
35–44	24 (24)	29 (30)
≥45	7 (7)	10 (10)
Sex (Male)	49 (49)	49 (51)
BMI (mg/kg2)		
<18.5 (underweight)	0	2 (2)
18.5 - <25 (normal)	44 (44)	44 (45)
25 - <30 (overweight)	33 (33)	28 (29)
≥ 30 (obese)	19 (19)	21 (22)
Blood pressure (mmHg)		
Normotensive (<130/85)	66 (66)	63 (65)
Elevated BP (≥130/≥85)	10 (10)	7 (7)
Systolic HTN (>130)	16 (16)	24 (25)
Diastolic HTN (>85)	7 (7)	3 (3)
Smoking		
Never	67 (67)	77 (79)
Past	22 (22)	9 (9)
Current	10 (10)	11 (11)
Social Economic Status		
Low (<Median = USD 138)	47 (47)	46 (47)
High (>Median = USD 138)	48 (48)	49 (51)
CD4 Cell count (cells/μl)	393 (57–729)	-
<350	34 (39)	-
>350	53 (61)	-
Viral Load (>1000copies/ml)	90 (90)	-
log_10_ VL	4.53 (3.2–5.8)	-

### Plasma lipid levels and dyslipidemia

Mean plasma lipid levels were similar between the HIV-infected and HIV-uninfected groups (Table 2). Mean TC was 96.6 mg/dl among HIV-infected participants and 95.3 mg/dl in the HIV-uninfected group (*p* = 0.77). There was also no difference in mean HDL, which was 30.9 mg/dl in the HIV-infected group and 31.6 mg/dl in the HIV-uninfected group (*p* = 0.69). The average TC: HDL ratio was normal in both groups: 3.2 among the HIV-infected and 3.1 among the uninfected (*p* = 0.22). Mean plasma lipid levels did not change after adjusting for storage-associated degradation.

**Table 2:** Mean plasma lipid values (non-fasting) for HIV-infected and uninfected participants

	HIV infected (*n* = 99)		HIV uninfected (*n* = 97)		
Lipid parameter (mg/dl)	Mean	(95% CI)	Mean	(95% CI)	*p*-value
TC	96.56	(90.33, 102.78)	95.3	(89.31, 101.29)	0.77
Plasma-HDL	30.93	(28.55, 33.31)	31.56	(29.54, 33.58)	0.69
TC:HDL ratio	3.24	(3.08, 3.40)	3.11	(2.97, 3.25)	0.22

The prevalence of dyslipidemia, defined as either TC >200 mg/dl or HDL <40 mg/dl, was 83.8% (*n* = 83) among HIV-infected individuals compared to 78.4% (*n* = 76) among HIV-uninfected individuals (Prevalence Ratio [PR] =1.07, 95% Confidence Interval [CI]: 0.9, 1.2; *p* = 0.33). In this cohort, observed dyslipidemia was characterized by low levels of HDL as we found no participants with elevated TC >200 mg/dl.

### Correlates of dyslipidemia

In univariate analysis, males had a higher prevalence of dyslipidemia (PR = 1.17; 95%CI: 0.93, 1.29; *p* = 0.4) while a 10-year increase in age showed a trend towards a significant association with dyslipidemia (PR = 0.05; 95% CI: -0.02, 0.10; *p* = 0.1). None of the other correlates investigated, including BMI, hypertension (including systolic and diastolic hypertension), smoking and socio-economic status, adjusted for age and gender were associated with dyslipidemia among both the HIV-infected and the HIV-uninfected.

Among HIV-infected participants, CD4 cell count was associated with dyslipidemia, with those having a CD4 cell count <350 cells/μl having a 30% higher likelihood of having dyslipidemia compared to those with CD4 cell count >350 cells/μl (PR = 1.31, 95% CI: 1.04, 1.41, *p* = 0.001). This remained unchanged upon adjusting for age, gender and viral load (aPR = 1.22, 95%CI: 1.08, 1.42, *p* = 0.01). In addition, those with a viral load ≥1000 copies/ml were 1.5-fold more likely to have dyslipidemia compared to those whose HIV-1 RNA was <1000 copies/ml, after adjusting for age, gender and CD4 cell count (aPR1.49; 95% CI: 1.14, 2.14; *p* = 0.02) (Table 3).

**Table 3:** Risk factors associated with dyslipidemia in HIV-infected and uninfected Kenyan adults

Characteristic	Normal	Dyslipidemia	Adjusted	95% CI^2^	*p*-value
	*n* = 37	*n* = 159	PR^1^		
Age (years)					
<25	7	14	1		0.1
25–34	19	86	1.23	0.84–1.62	
35–44	9	44	1.25	0.84–1.65	
≥45	2	15	1.32	0.86–1.79	
Gender					
male	16	81	1.06	0.92–1.20	0.4
female	21	78	1	Ref	
BMI (mg/kg2)^a^					
<18.5 (underweight)	1	1	0.78	−0.05 - 1.61	
18.5 - <25 (normal)	19	69	1	Ref	0.57
25 - <30 (overweight)	9	52	1.11	0.93–1.28	
≥30 (obese)	7	33	1.05	0.85–1.26	
Blood Pressure					
Normotensive	25	104	1	Ref	0.95
Elevated BP (≥130/≥85)	4	13	0.93	0.66–1.19	
systolic HTN^3^ (>130)	6	34	1	0.83–1.19	
diastolic HTN^3^ (>85)	2	8	1	0.72–1.30	
Smoking					
Never	27	117	1	Ref	
Ever	10	42	0.95	0.77–1.12	0.56
SES^4^(Ksh 12,000)^b^					
Above median	19	78	1		
Below median	17	76	1.02	0.88–1.16	0.74
HIV Status					
HIV-	21	76	1		
HIV+	16	83	1.08	0.94–1.23	0.24
Viral Load (copies/ml)					
<1000	4	5	1	Ref	
>1000	12	78	1.56	1.14–2.14	**0.02**
CD4 Cell count (cells/μl)^c^					
>350	13	40	1	Ref	
<350	1	33	1.31	1.08–1.42	**0.01**

^a^N = 191

^b^N = 190

^c^N = 87

^1^Prevalence Ratio

^2^95% Confidence Interval

^3^Hypertension

^4^Socioeconomic status

## Discussion

In this non-fasting cohort comparing ART naïve HIV-infected individuals to HIV-uninfected individuals, the proportion of individuals with dyslipidemia was high (>81.1%) irrespective of HIV infection status. Among HIV-infected participants, high viral load was significantly associated with increased risk of dyslipidemia, even after adjusting for age and gender, and lower CD4 cell count was also associated with dyslipidemia. Although there was no significant difference in prevalence of dyslipidemia comparing HIV-infected and HIV-uninfected groups, there was a slightly higher proportion of HIV-infected with low HDL (83.8%) compared to HIV-uninfected (78.4%). Although statistically insignificant, the total cholesterol was also higher among the HIV-infected relative to the HIV-uninfected. This is in keeping with other studies which have found high total cholesterol levels, high triglyceride levels and low HDL levels among similar ART naïve populations in Cameroon, Nigeria and Tanzania [9, 10, 19, 20].

The interplay between HIV infection, cardiovascular disease and dyslipidemia is complex and not fully understood. The HIV virus is associated with vascular structural and functional alterations [21]. One molecular theory of HIV-induced endothelial dysfunction is that HIV synthesizes Tat, a transcriptional protein secreted by HIV-infected cells that increases the expression of adhesion molecules and induces apoptosis of endothelial cells, which allows the penetration of plasma lipids (low density lipoproteins) into the sub-endothelial space where they undergo oxidation. The adhesive proteins and inflammatory cytokines promote the recruitment of monocytes into the intima where they transform into macrophages and foam cells by engulfing the accumulated lipids [22]. Upon their apoptotic death, these lipid-rich macrophages contribute toward the formation of the necrotic lipid core that is the hallmark of advanced atherosclerotic lesions [22–24]. The significant association between viral load and risk of dyslipidemia, independent of age and gender, is in keeping with the proposed biological mechanism described above. A higher viral load would be associated with increased adhesive proteins and inflammatory cytokines with depletion of anti-inflammatory HDL. Several studies that have shown LDL as a major modifiable cardiovascular risk factor have conversely shown an inverse association between HDL levels and cardiovascular events [25].

Similarly, association between a low CD4 cell count and higher prevalence of dyslipidemia is in keeping with the HIV-induced inflammation mechanism. Lower immune function is associated with increased inflammation and thus it is expected that HIV-infected individuals would have a higher risk of developing dyslipidemia compared to HIV-uninfected individuals. This process may be limited in an immune competent HIV-infected individual, similar to what might be observed in a HIV-uninfected individual. A possible explanation for the lack of difference in dyslipidemia between the HIV-infected ART naïve and the HIV uninfected in our study population could thus be high-functioning immune systems. While the median CD4 count in our cohort was 393 cells/μl, most ART naïve cohorts demonstrating significant difference between HIV-infected and uninfected individuals have had low CD4 counts (≤200cells/μl) on average among the HIV uninfected [9, 10, 26].

The lack of difference in dyslipidemia prevalence could also be attributed to a relatively high prevalence of dyslipidemia among the general Kenyan population. Recently, the Africa Middle East Cardiovascular Epidemiological (ACE) Study assessing cardiovascular risk factors in the general population found dyslipidemia prevalence at 70% in Kenya mostly marked by low HDL levels compared to 45% in Cameroon where 2 studies had previously found significantly higher dyslipidemia in ART naïve HIV-infected individuals relative to HIV- uninfected [27, 28]. With the high prevalence of dyslipidemia among HIV-uninfected individuals (78%), we had a limited ability to show a difference between HIV-infected and uninfected individuals.

The use of a viral load cut-off of 1000 copies/ml and CD4 cell count of 350 cells/μl for association with dyslipidemia is relevant because of their clinical relevance in defining significant viremia and eligibility for ART initiation respectively in primary HIV care settings in Sub Saharan Africa at the time. Using these cut offs, 39% of our participants would have been eligible for initiation of ART and would have been at an increased risk of a worsening lipid profile, as long term use of ART is associated with an atherogenic lipid profile; characterized by an increase in triglycerides, total cholesterol and low density lipoprotein (LDL) cholesterol levels and a modest rise in HDL [25, 29].

HIV serodiscordant couples are an optimal study population as they are presumed to have similar unmeasured dietary and environmental confounders that would affect their lipid levels. This and the fact that we had accurate CD4 and HIV-1 viral load measures compared to other studies in similar settings that have used routine CD4 and HIV-1 viral load measures whose measurement may not be consistent over time were significant study strengths. However, there were several limitations. Our mean lipid levels were low compared to similar populations previously described [10], which could be an artifact of the extended period of storage (up to 7 years) prior to lipid measurement. Although the mean plasma lipid levels and prevalence of dyslipidemia did not vary upon adjustment for storage, the degree to which storage truly affected the lipid levels in our sample remains unclear and could affect the reported prevalence of dyslipidemia. The rate of degradation would be expected to be similar among HIV-infected and uninfected, leading to a bias towards the null when comparing the two groups. Another limitation is that we used non-fasting plasma samples and therefore could not assess triglyceride and LDL levels, which would have added robustness to our description of dyslipidemia in this population.

## Conclusion

In summary, our results demonstrate a high prevalence of dyslipidemia characterized by low-HDL and associated with a high viral load and low CD4 cell count. This suggests that screening for dyslipidemia in ART naïve individuals, even in a non-fasting state, is still important as it would identify HIV-infected individuals at elevated risk of developing an even higher TC: HDL ratio upon ART initiation and may help inform the choice of their ART drug regimen.

## Abbreviations

aPR: Adjusted prevalence ratio
ART: Antiretroviral treatment
BMI: Body Mass Index
ELISA: Enzyme-linked immunosorbent assay
HDL: High density lipoprotein cholesterol
HTN: Hypertension
IQR: Interquartile range
KNH-UoN: Kenyatta National Hospital/University of Nairobi
MI: Myocardial Infarction
SES: Socio-economic status
TC: Total cholesterol
VCT: Voluntary counseling centers
